# P03 A qualitative exploration of antimicrobial stewardship and resistance education in undergraduate healthcare professional programmes

**DOI:** 10.1093/jacamr/dlac004.002

**Published:** 2022-02-16

**Authors:** Aoife Fleming, Ornaith Keane, Long Nguyen, Dylan Burke, Teresa M Barbosa

**Affiliations:** Pharmaceutical Care Research Group, School of Pharmacy, University College Cork, Ireland; Pharmacy Department, Mercy University Hospital, Cork, Ireland; Pharmaceutical Care Research Group, School of Pharmacy, University College Cork, Ireland; Pharmaceutical Care Research Group, School of Pharmacy, University College Cork, Ireland; Pharmaceutical Care Research Group, School of Pharmacy, University College Cork, Ireland; Pharmaceutical Care Research Group, School of Pharmacy, University College Cork, Ireland

## Abstract

**Background:**

Education of undergraduate healthcare professionals (HCPs) on antimicrobial stewardship (AMS) and antimicrobial resistance (AMR) is recommended as an important strategy to prepare future HCPs for their roles in these areas.^1^ There is limited qualitative research investigating the views of clinical and academic staff involved in the teaching of undergraduate HCP students on current AMS/AMR education practices and curricula.

**Objectives:**

To explore the views and experiences of clinical and academic staff involved in HCP and public health programmes on AMS and AMR education.

**Methods:**

A qualitative study using semi-structured interviews was conducted in October to December 2020. The interview participants were purposively sampled to recruit clinical or academic staff involved in education of students on undergraduate HCP programmes (dentistry, medicine, nursing and midwifery, pharmacy) and public health programmes in an Irish university. Participants were asked to outline their views on current teaching and assessment methods, use of AMS competency frameworks and potential areas for improvement. Interview recordings were conducted online, the recordings were transcribed verbatim, and transcripts analysed by thematic analysis. Ethical approval was obtained, and all participants provided written informed consent.

**Results:**

Interviews were conducted with 15 participants (10 female). A range of disciplines were represented as outlined in the methods. The main interview themes included the challenge of balancing curriculum priorities and capacity in HCP programmes, the importance of ensuring student understanding of AMS/AMR principles, and patient centred application of knowledge. Current teaching and assessment methods were found to vary between the HCP programmes with a mix of didactic lectures and workshops, and assessment ranging from written exams and multiple choice questions to observed structured clinical exams. Very few participants reported educating different HCP students together. Participants were asked to review an antimicrobial education competency framework,^2^ with most participants believing that this would improve the structure of AMS education, ensuring that key learning outcomes would be addressed in the curriculum. The importance of preparing students for interprofessional practice in AMS in the future was identified as a key theme, with participants highlighting the importance of interprofessional education opportunities to foster communication and teamwork skills in AMS at an early stage.

**Conclusions:**

The importance of AMS and AMR education in HCP programmes was reported in this study, however the main challenge is limited curricular space in busy HCP programmes. Education may be improved by aligning AMS education with an appropriate competency framework. Enhancement of undergraduate interprofessional education opportunities, to prepare students for interprofessional AMS in future practice, is recommended.
